# Dynamic event-based optical identification and communication

**DOI:** 10.3389/fnbot.2024.1290965

**Published:** 2024-02-12

**Authors:** Axel von Arnim, Jules Lecomte, Naima Elosegui Borras, Stanisław Woźniak, Angeliki Pantazi

**Affiliations:** ^1^fortiss GmbH, Neuromorphic Computing, Munich, Germany; ^2^IBM Research Zurich, Rüschlikon, Switzerland; ^3^Neural Systems and Computation, University of Zurich and ETH Zurich, Zürich, Switzerland

**Keywords:** neuromorphic computing, event-based sensing, optical camera communication, optical flow, identification

## Abstract

Optical identification is often done with spatial or temporal visual pattern recognition and localization. Temporal pattern recognition, depending on the technology, involves a trade-off between communication frequency, range, and accurate tracking. We propose a solution with light-emitting beacons that improves this trade-off by exploiting fast event-based cameras and, for tracking, sparse neuromorphic optical flow computed with spiking neurons. The system is embedded in a simulated drone and evaluated in an asset monitoring use case. It is robust to relative movements and enables simultaneous communication with, and tracking of, multiple moving beacons. Finally, in a hardware lab prototype, we demonstrate for the first time beacon tracking performed simultaneously with state-of-the-art frequency communication in the kHz range.

## 1 Introduction

Identifying and tracking objects in a visual scene has many applications in sports analysis, swarm robotics, urban traffic, smart cities, and asset monitoring. Wireless solutions have been widely used for object identification, such as RFID (Jia et al., 2012) or more recently Ultra Wide Band (ITU, 2006), but these do not provide direct localization and require meshes of anchors and additional processing. One efficient solution is to use a camera to detect specific visual patterns attached to the objects.

This optical identification is commonly implemented with frame-based cameras, either by recognizing a spatial pattern in each single image—for instance for license plate recognition (Du et al., 2013)—or by reading a temporal pattern from an image sequence (von Arnim et al., 2007). The latter is resolution-independent, since the signal can be reduced to a spot of light, enabling for much faster frame frequencies. It can be implemented with near-infrared blinking beacons that encode a number in binary format, similarly to Morse code, to identify assets like cars or road signs. But frame-based cameras, even at low resolutions, impose a hard limit on the beacon's frequency (in the 10^2^ Hz order of magnitude). This technique is known as Optical Camera Communication (OCC) and has been developed primarily for communication between static objects (Cahyadi et al., 2020).

Identifying static objects is possible with OCC as discussed before, but in applications such as asset monitoring on a construction site, it is also important to track dynamically moving objects. OCC techniques potentially enable simultaneous communication with, and tracking of, beacons. However, two challenges arise in the presence of relative movements: filtering out the noise and tracking the beacons' positions. Increasing the temporal frequency of the transmitted signal, since noise has lower frequencies than the beacon's signal, addresses this problem. Nevertheless, current industrial cameras do not offer a satisfying spatio-temporal resolution trade-off. Biologically-inspired event cameras, operating with temporally and spatially sparse events, achieve pixel frequencies on the order of 10^4^ Hz and can be combined with Spiking Neural Networks (SNNs) to build low-latency neuromorphic solutions. They capture individual pixel intensity changes extremely fast rather than full frames (Perez-Ramirez et al., 2019). Early work combined the fine temporal and spatial resolution of an event camera with blinking LEDs at different frequencies to perform visual odometry (Censi et al., 2013). Recent work makes use of these cameras to implement OCC with smart beacons and transmit a message with the UART protocol (Wang et al., 2022), delivering error-free messages of static beacons at up to 4 kbps indoors and up to 500 bps at 100 m distance outdoors with brighter beacons, but without tracking. This paper, combined with the tracking approach presented in von Arnim et al. (2007), are the baseline of our work. The Table 1 summarizes the properties of the mentioned methods.

**Table 1:** Characteristics of existing identification methods.

**Method**	**Type of camera**	**Data throughput (bps)**	**Tracking**
von Arnim et al. (2007)	Frame-based	250	Yes
Perez-Ramirez et al. (2019)	Event-based	500	No
Wang et al. (2022)	Event-based	500	No
Censi et al. (2013)	Event-based	Identification only	Yes
Ours	Event-based	2500	Yes

The table presents existing optical camera communication solutions, using frame-, or event-based cameras.

On the tracking front—to track moving beacons in our case—a widely used technique is optical flow (Chen et al., 2019). Model-free techniques relying on event cameras for object detection have been implemented (Barranco et al., 2018; Ojeda et al., 2020). To handle the temporal and spatial sparsity of an event camera, a state-of-the-art deep learning frame-based approach (Teed and Deng, 2020) was adapted to produce dense optical flow estimates from events (Gehrig et al., 2021). However, a much simpler and more efficient solution is to compute sparse optical flow with inherently sparse biologically-inspired SNNs (Orchard et al., 2013), also considering network optimisation and improved accuracy (Schnider et al., 2023).

In this paper, we propose to exploit the fine temporal and spatial resolution of event cameras to tackle the challenge of simultaneous OCC and tracking, where the latter is based on the optical flow computed from events by an SNN. We evaluate our approach with a simulated drone that is monitoring assets on a construction site. We further introduce a hardware prototype comprising a beacon and an event camera, which we use for demonstrating an improvement over state-of-the-art OCC range. To our knowledge, there is no method combining event-based OCC with tracking to identify moving targets. Furthermore, we beat the transmission frequency of our baseline.

## 2 Materials and methods

**Figure 1:**
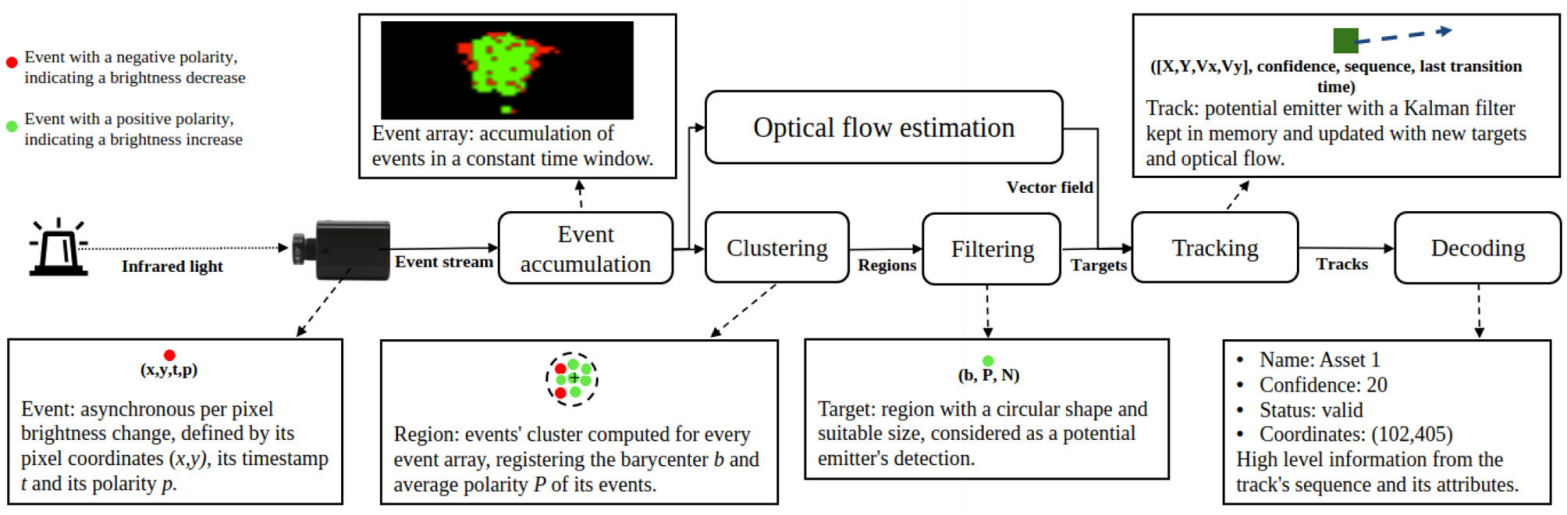
Architectural diagram of our system. The beacon's light is detected by the sensor as events. Events are processed to track the beacons and further decode the transmitted messages. The event array block shows a snapshot of recorded events.

The system that we propose is composed of an emitter and a receiver. The former is a beacon emitting a temporal pattern (a bit sequence) with near infrared light (visible to cameras, but not to humans), attached to the object to be identified and tracked. The receiver component is an event-based camera connected to a computer which, in turn, executes decoding and tracking algorithms. The receiver part comprises algorithmic components for clustering and tracking for which an SNN calculates optical flow. The entire process, from low-level event-processing to high-level (bit-)sequence-decoding, is schematically depicted in Figure 1. This figure also introduces specific terms that are used throughout the rest of this paper.

The proposed system is a hybrid of a neuromorphic and an algorithmic solution. It follows a major trend in robotics to exploit the rich capabilities of neural networks, which provide sophisticated signal processing and control capabilities (Li et al., 2017). Simultaneously, to handle the temporal and noisy nature of the real-world signals, neural networks can be extended to handle time delays (Jin et al., 2022), or to include stages with Kalman filtering (Yang et al., 2023), leading to a synergy between neural networks and classic algorithms. Our system follows a similar approach and subsequent paragraphs describe its components.

### 2.1 Event-based communication

**Figure 2:**
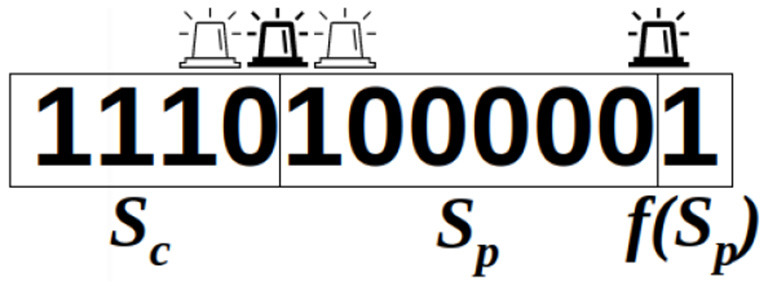
A valid sequence, decoded from blinking transitions.

The emitter is synchronously transmitting, with a blinking pattern, a binary sequence *S* that consists of a start code *S*_*c*_, a data payload (identification number) *S*_*p*_ and a parity bit *f*(*S*_*p*_), where *f* returns 1 if *S*_*p*_ has an even number of ones, or 0 otherwise. The start code and the parity bit delimit the sequence and confirm its validity, as illustrated in Figure 2. On the receiver side, the event camera asynchronously generates events upon pixel brightness changes, which can be caused by either a change in the beacon's signal or visual noise in the scene. The current state of the beacon (**on** or **off**) cannot be detected by the sensor. Rather, the sensor detects when the beacon transitions between these states. The signal frequency being known, the delay between those transitions gives the number of identical bits emitted. In comparison to a similar architecture with a frame-based camera (200 Hz frame rate) (von Arnim et al., 2007), our setup relies on an event camera and a beacon blinking in kHz frequency, allowing for a short beacon decoding time, better separation from noise and easier tracking since beacon's motions are relatively slower.

As the start code *S*_*c*_ is fixed and the identification number *S*_*p*_ is invariable per beacon, the parity bit *f*(*S*_*p*_) remains the same from one sequence to the next. As a result, once the beacon parameters are set, it repeatedly emits the same 11-bit fixed-length frame. The decoding of the transmitted signal exploits these two transmission characteristics. As the cameras do not necessarily pick up the signal exactly from the start code, 11 consecutive bits are stored in memory. If the signal is received correctly, these 11 bits constitute a full sequence. Once this sequence of 11 bits is recovered, it is necessary to search for the subsequence of four bits corresponding to the start code *S*_*c*_ (marked below in bold), which enables to recover a complete sequence through bit rotation:

- Reception of 11 successive bits: 0 0 0 0 0 1 **1 1 1 0** 1
- Sequence reconstruction after start code detection: **1 1 1 0** 1 0 0 0 0 0 1

### 2.2 Object tracking

Beacons isolated by the clustering and filtering steps described in Figure 1 are called targets. These are instant detections of the beacons. But these need be tracked in order to extract the blinking code that they produce. The tracked targets are called tracks. They hold a position (estimated or real), the history of state changes (ons and offs) and meta information like a confidence value. Tracks are categorized with types that can change over time. They can be:

- new: the target cannot be associated with any existing track: create a new track
- valid: the track's state change history conforms to the communication protocol
- invalid: the track's state change history does not conform to the communication protocol (typically noise or continuous signals like solar reflections). Note that a track can change from invalid to valid if it's confidence value rises (detailed later).

#### 2.2.1 Clustering

Camera events are being accumulated in a time window and clustered with the Density-Based Spatial Clustering of Applications with Noise (?), chosen to get rid of noisy, isolated events and to retrieve meaningful objects from the visual scene. Such clusters are filtered according to:


(1) $$\begin{array}{l} \frac{N_{e}}{\pi \times |b-d{|}^{2}}>r,N_{e}\in \left\{N_{\text{min}};N_{\text{max}}\right\} \end{array}$$

where *N*_*e*_ is the number of events in the cluster, |*b*−*d*| the Euclidean distance between the cluster's barycenter *b* and *d* its most distant event, *r* a shape ratio, and *N*_min_ and *N*_max_ the minimal/maximal emitter size in pixel. The shape ratio is a hyperparameter. In our setup, it characterizes the roundness of the cluster, since we are looking for round beacons. It can be adapted to other shapes if beacons need be flatter for example. Experimentally, the shape ratio *r* turned out to play a crucial role in the communication's accuracy: limiting the detection to high ratios (from 0.8 to 0.99) gave the best results. The minimal target size *N*_min_ of target must also be carefully set to be able to detect beacons, but small values also imply filtering less noise and having to process more clusters. Depending on the scenario distances, values from 5 to 30 events were chosen.

We reduce the remaining clusters to their barycenter, size and their polarity and call these “targets.” The polarity of a target *P* is given by $P=\left(\underset{i}{\sum }p_{i}\right)/N_{e}$ where *p*_*i*_=1 for a positive polarity and −1 for a negative one for each event *i*.

#### 2.2.2 Event-based optical flow

Event-based optical flow is calculated by a neural network and processed by the remaining algorithmic beacon tracking pipeline. We introduce it as a given input in the main tracking algorithm presented in the next section.

Optical flow is computed from the same camera and events that are used for decoding, and delivers a sparse vector field for visible events with velocity and direction.

We implemented an SNN architecture with Spiking Neural Units (SNUs) (Woźniak et al., 2020) and extended the model with synaptic delays that we call ΔSNU. Its state equations are:


(2) $$\begin{array}{ll} s_{t} & =g\left(Wd_{\Delta }\left(x_{t}\right)+l\left(\tau \right)s_{t-1}\left(1-y_{t-1}\right)\right) \end{array}$$

(3) $$\begin{array}{ll} y_{t} & =h\left(s_{t}-v_{\text{th}}\right), \end{array}$$

where *W* are the weights, *v*_th_ is a threshold, *s*_*t*_ is the state of the neuron and *l*(τ) its decay rate, *y*_*t*_ is the output, *g* is the input activation function, *h* is the output activation function, and *d* is the synaptic delay function. The delay function *d* is parameterized with a delay matrix Δ that for each neuron and synapse determines the delay at which spikes from each input *x*_*t*_ will be delivered for the neuronal state calculation.

**Figure 3:**
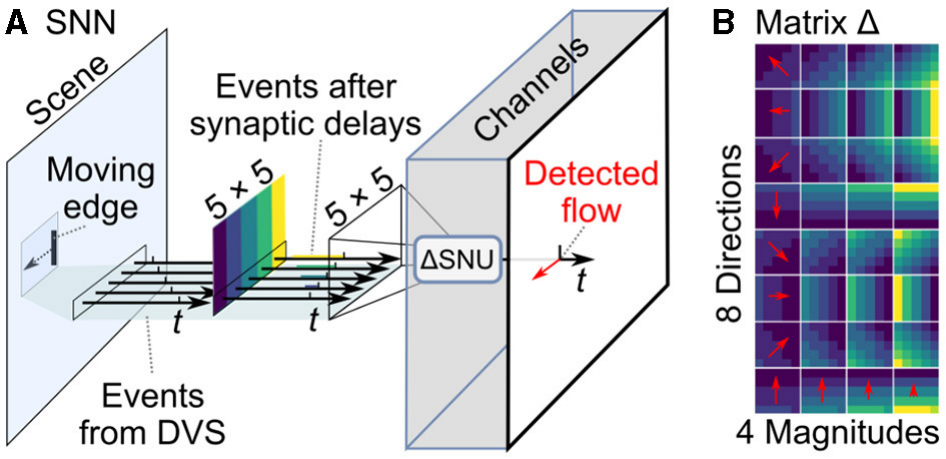
SNN for sparse optical flow. **(A)** Events from camera at each input location are processed by 32 ΔSNU units, each with specific synaptic delays. **(B)** The magnitudes of synaptic delays are attuned to 8 different movement angles (spatial gradient of delays) and 4 different speeds (different magnitudes of delays), schematically indicated by red arrows.

Optical flow is computed by a CNN with 5 × 5 kernels, illustrated in Figure 3A. Each ΔSNU is attuned to the particular direction and speed of movement through its specific synaptic delays, similarly to (Orchard et al., 2013). When events matching the gradient of synaptic delays are observed, a strong synchronized stimulation of the neuron leads to neuronal firing. This results in sparse detection of optical flow. The synaptic delay kernels are visualized in Figure 3B. We use 8 directions and 4 magnitudes, with the maximum delay period corresponding to 10 executions of the tracking algorithm. Weights are set to one and *v*_th_ = 5. The parameters were determined empirically so as to yield the best tracking results. Decreasing the threshold *v*_th_ yields faster detection of optical flow, but increases the false positive spikes. Increasing the number of detected directions and magnitudes theoretically provides more accurate estimation of the optical flow. However, in practice it results in false positive activation of neurons detecting similar directions or magnitudes unless *v*_th_ is increased at the expense of increased detection latency.

#### 2.2.3 Tracking

**Figure 4:**
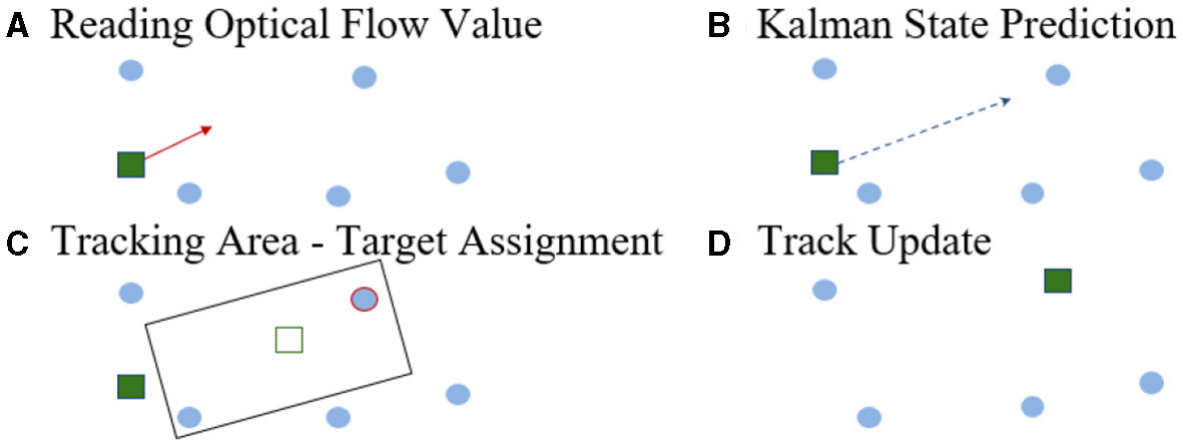
Tracking steps. **(A)** Reading optical flow (red arrow) at the track's location. **(B)** Prediction of the Kalman state via the track's location and the optical flow value. **(C)** Tracks are assigned to a target in its oriented neighborhood, based upon the track's motion. **(D)** The track's state, its size and its polarity are updated with the paired target's properties.

Targets are kept in memory for tracking over time and are then called tracks. A Kalman filter is attributed to each track and updated for every processed time window, as depicted in Figure 4. A Kalman filter is needed to estimate the position of a track from the last measured one and when is not visible, either because of an occlusion, or simply because it transitioned to **off**. We use the estimated position's optical flow value to draw a search window in which a target is looked for. Similarly to Chen et al. (2019), predicted tracks' states are matched to detected targets to minimize the L1-norm between tracks and targets. Unmatched targets are registered as new tracks.

#### 2.2.4 Identification

A matched track's sequence is updated using the target's mean event polarity *P*.

- If *P* ≥ 0.5 then the beacon is assumed to have undergone an **on** transition. We add *n* = (*t*_*c*_ − *t*_*t*_)/*f*_beacon_ zeros to the binary sequence where *t*_*c*_ is the current timestamp, *t*_*t*_ is the stored last transition timestamp and *f*_beacon_ is the beacon blinking frequency and set *t*_*t*_ = *t*_*c*_.
- If *P* ≤ −0.5 then the beacon is assumed to have undergone a transition to the **off** state. Likewise, we add *n* ones to the binary sequence.
- Otherwise, the paired beacon most likely has not undergone a transition but just moved.

Similarly to von Arnim et al. (2007), a confidence value is incremented or decremented to classify tracks as new, valid or invalid, as illustrated in Algorithm 1. Indeed, noise can pass clustering filters but will soon be invalidated as its confidence will never rise. To correct for errors (for instance due to occlusions), the confidence increments are larger than decrements. When a track's sequence is long enough to be decoded, it is declared valid if it complies to the protocol and maintains the same payload (if this track was previously correctly recognized). New tracks have an initial confidence value ≤ confidence_max_. These values have been experimentally set to optimize for our protocol and an expected mean occlusion duration. They can be adapted for expected longer **off** states or longer occlusions. Though, the level of track robustness to occlusion and its "stickyness" have to be balanced. Indeed, higher confidence thresholds lead to a longer detection time and also a longer time to become invalid. A clean up of tracks having been invalid for too long is necessary in all cases to save memory. This is done with a simple threshold (confidence_min_) or a time out (delay_max_) mechanism. These hyper-parameters were tuned experimentally, and we set them to confidence_min_ = 0, confidence_max_ = 20, and the initial confidence to 10.

**Algorithm 1:**
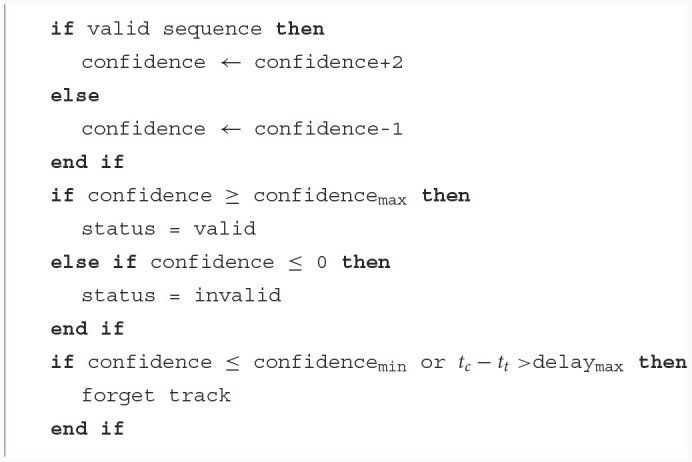
Track classification with a confidence system.

To ensure real-time execution, the tracking occurs at a lower frequency, while the decoding occurs at the emitter frequency. To achieve this, we only accumulate events in the surrounding of existing tracks, and tracks' sequences are updated accordingly.

#### 2.2.5 Computational performance

**Figure 5:**
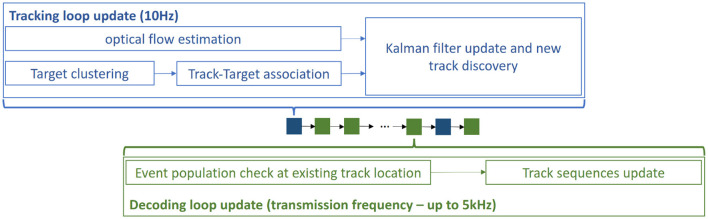
Decoupled loops: The tracking loop has a much lower and fixed frequency to maintain efficiency, while the decoding loop has the same frequency as the emitter to be able to decode the received signal.

The hardware event-based camera can detect up to millions of events per second, where many of them may correspond to noise, especially in outdoor and moving camera scenarios. The tracking algorithm, based on a neuronal implementation of optical flow and on a clustering algorithm with a square complexity on the number of events, is computational much more demanding than the decoding algorithm. Therefore, to ensure real-time performance, both loops have been decoupled, so that the tracking is updated at a lower frequency than the decoding, as illustrated in Figure 5. In this implementation, tracking steps described above occur at 10 Hz, while the decoding happens at up to 5kHz. The relative motion of tracked objects in the visual scene being slow compared to the communication event rate, the tracking update rate is sufficient. To ensure a working communication, the decoding algorithm must be fast and computationally inexpensive to match the emitter frequency.

## 3 Results

### 3.1 Static identification

**Figure 6:**
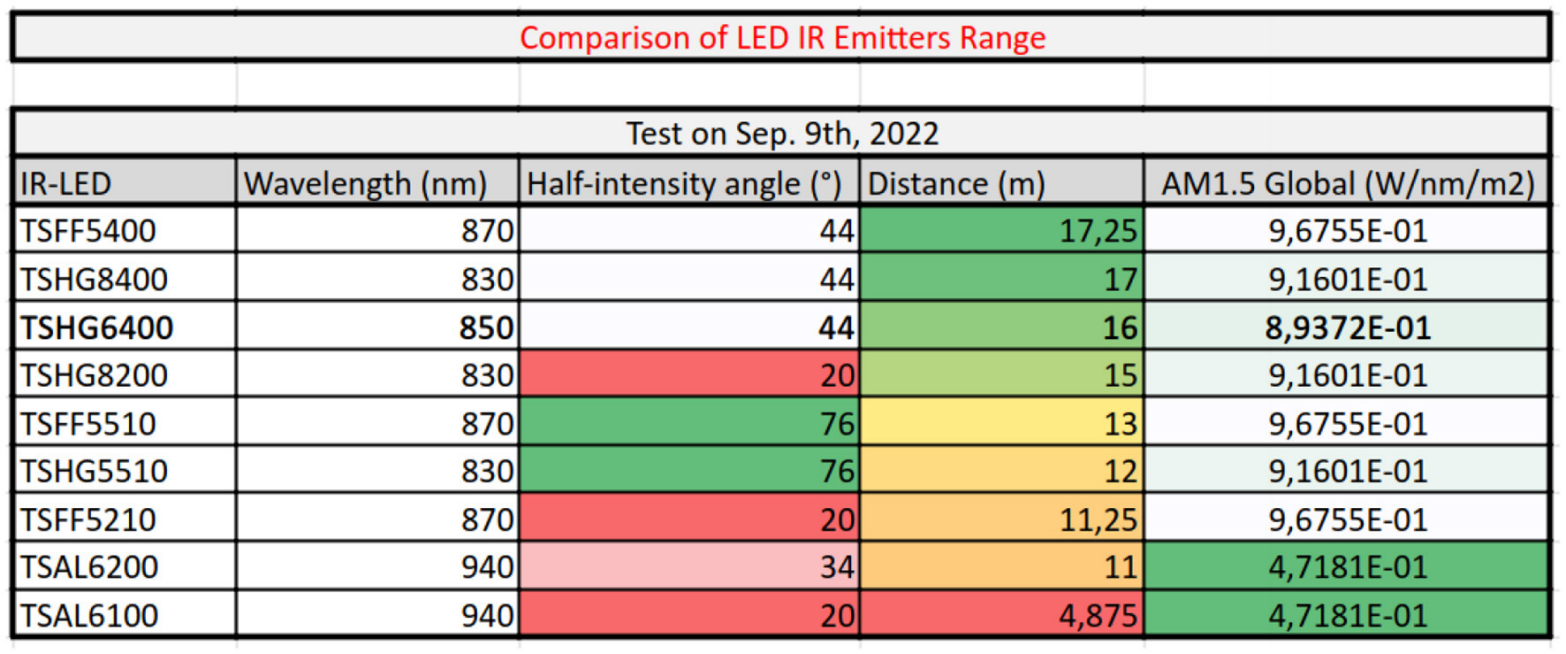
LED wavelength benchmark: The range of LEDs with varying wavelength and half-intensity angle was experimentally determined. AM1.5 Global is the solar integrated power density. The final choice of 850 nm ensures a good trade-off between detection range and outdoor solar irradiance.

**Figure 7:**
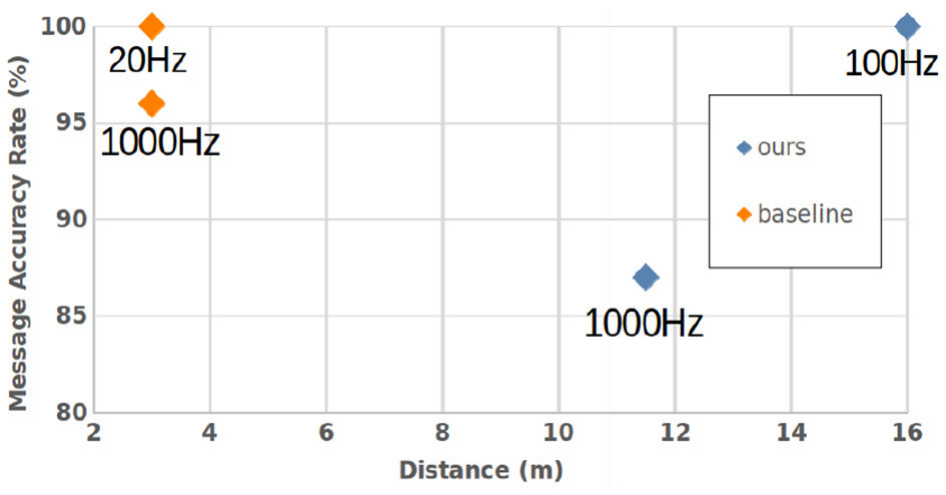
Static OCC performance: MAR for increasing beacon frequencies in comparison with the state-of-the-art baseline (Wang et al., 2022). Results were obtained at a 50 cm distance.

**Figure 8:**
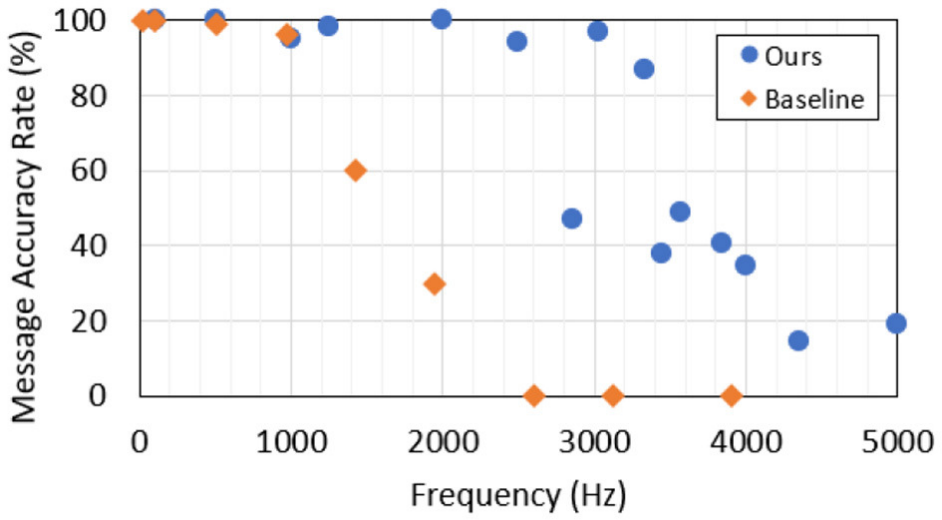
Static OCC performance: MAR for increasing beacon distance to the camera in comparison with the state-of-the-art baseline (Wang et al., 2022).

Our hardware beacon has four infrared LEDs (850 nm) and an ESP32 micro-controller to set the payload *S*_*p*_ = 42 and the blinking frequency. A study was conducted to find the optimal wavelenght where the LEDs must be detected as far as possible in an outdoor use case, as described in Figure 6. To receive the signal, we used a DVXplorer Mini camera, with a resolution of 640 × 480 and a 3.6 mm focal length lens. In a static indoor setup, the hardware event camera enables us to achieve high data transmission frequencies, plotted in Figure 7. The metric is the Message Accuracy Rate (MAR): the percentage of correct 11-bit sequences decoded from the beacon's signal during a recording. The MAR stays over 94 % up to 2.5 kHz, then decreases quickly, due to the temporal resolution of the camera. Using a 16 mm focal length lens we could identify the beacon at a distance of 11.5 m indoors, with 87 % MAR and a frequency of 1 kHz and obtained 100 % MAR at 100 Hz at 16 m—see Figure 8.

A special note has to be made regarding the range. Results are given here for information purpose. The range cannot really be considered a benchmarking parameter because it depends essentially on the beacon signal power and on the camera lens. To improve the detection and MAR at a longer range, adding LEDs to the beacon, or choosing a zooming lens, are good solutions. So this is basically an implementation choice.

### 3.2 Dynamic identification

**Figure 9:**
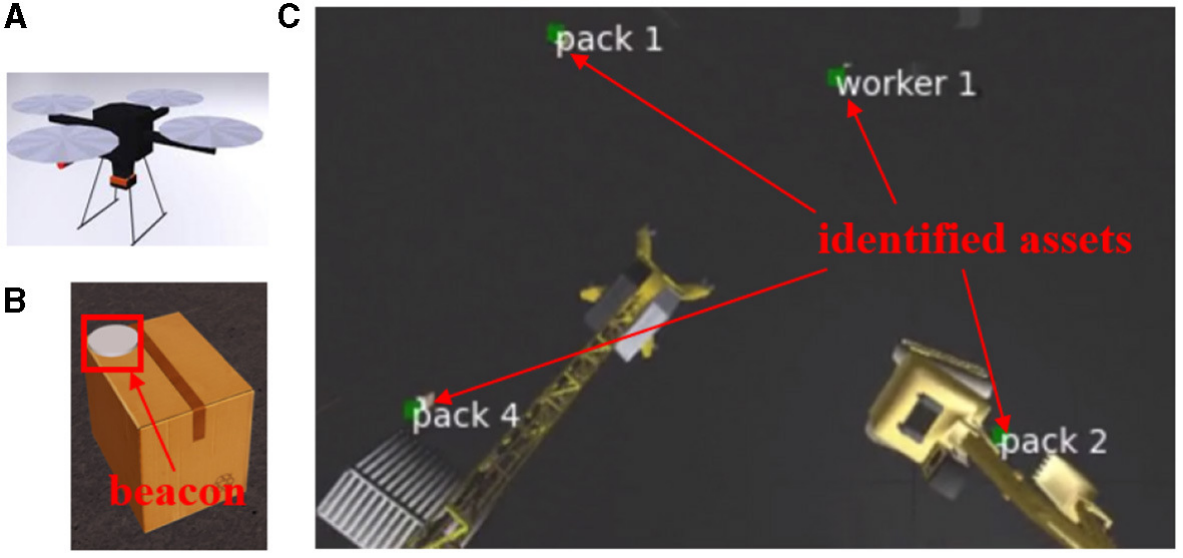
Simulation setup. **(A)** Hector quadrotor. **(B)** Example asset. **(C)** The drone's point of view with decoding results.

To evaluate our identification approach in a dynamic setup, where tracking is required, a simulated use case was developed in the Neurorobotics Platform (Falotico et al., 2017). A Hector drone model, with an on-board event camera plugin (Kaiser et al., 2016), flies over a construction site with assets (packages and workers) to be identified and tracked. These are equipped with blinking beacons. The drone follows a predefined trajectory and the scene is captured from a bird's eye view—see Figure 9. A frame-based camera with the same view is used for visualization. Noise is simulated with beacons of different sizes blinking randomly. For varying drone trajectories, assets were correctly identified at up to 28 m, with drone speeds up to 10 m/s (linear) and 0.5 radian/s (self rotational). Movements were fast, relative to the limited 50 Hz beacon frequency imposed by the simulator. A higher MAR was obtained with a Kalman filter integrating optical flow (Section 2.2.3) than without it—see Table 2. MAR and Bit Accuracy Rate (BAR) are correlated in simulation because they drop together only upon occlusion. Finally, we conducted hardware experiments where a beacon was moved at 2 m/s reaching a 94 % BAR at 5m and a 87 % BAR at 16m. This shows that our system enables accurate identification and data transmission even with moving beacons, which, to our knowledge, is beyond the state-of-the-art.

**Table 2:** Identification performance with moving beacons.

**Setup**	**Rate**	**Range**	**MAR**	**BAR**
Simulation with optical flow	50 Hz	28 m	74 %	75 %
Simulation without optical flow	50 Hz	28 m	71 %	72 %
Hardware	2,000 Hz	5 m	65 %	94 %
Hardware	2,000 Hz	16 m	27 %	87 %

## 4 Discussion

We propose a novel approach for identification that combines the benefits of event-based fast optical communication and signal tracking with spiking optical flow. The approach was validated in a simulation of drone-based asset monitoring on a construction site. A hardware prototype setup reached state-of-the-art optical communication speed and range. We propose the first—to the best of our knowledge—system to identify fast moving, variable beacons with an event camera, thanks to our original tracking approach. Event-based camera, thanks to their extremely low pixel latency, do outperform OCC based on frame grabbers by orders of magnitude. This enables beacon signal frequencies up to 5 kHz, which in turn, enables for their more robust tracking, since their relative movement is slow between two LED state transitions. Nevertheless, tracking is still necessary for beacons moving fast and that is where this work goes beyond (Wang et al., 2022), which assumes null or negligible beacon movement.

Further research includes the port of optical flow computation to neuromorphic hardware and the full port of the system onto a real drone, for real world assessment. Although the current work is mainly algorithmic with optical flow realized in a spiking neural network, this paper proves that it is very efficient. Now, as this mixes two computing paradigms (algorithmics and spiking neural networks), it will entail having two computing devices on board a real drone. Another research direction of ours is thus to investigate a full spiking implementation, so as to carry only neuromorphic hardware on board.

## Data availability statement

The raw data supporting the conclusions of this article will be made available by the authors, without undue reservation.

## Author contributions

AA: Conceptualization, Formal analysis, Funding acquisition, Investigation, Methodology, Project administration, Resources, Supervision, Validation, Writing – original draft, Writing – review & editing. JL: Data curation, Formal analysis, Investigation, Methodology, Software, Validation, Visualization, Writing – original draft, Writing – review & editing. NB: Formal analysis, Investigation, Methodology, Software, Writing – review & editing. SW: Conceptualization, Formal analysis, Investigation, Methodology, Supervision, Validation, Writing – original draft, Writing – review & editing. AP: Conceptualization, Formal analysis, Funding acquisition, Project administration, Resources, Supervision, Validation, Writing – review & editing.
